# Cyanogenic Glycoside Analysis in American Elderberry

**DOI:** 10.3390/molecules26051384

**Published:** 2021-03-04

**Authors:** Michael K. Appenteng, Ritter Krueger, Mitch C. Johnson, Harrison Ingold, Richard Bell, Andrew L. Thomas, C. Michael Greenlief

**Affiliations:** 1 Department of Chemistry, University of Missouri, Columbia, MO 65211, USA; mkappenteng@mail.missouri.edu (M.K.A.); rmkqm2@mail.umkc.edu (R.K.); mcjohnson0989@gmail.com (M.C.J.); hgidn9@mail.missouri.edu (H.I.); 2 Department of Chemistry, Truman State University, Kirksville, MO 63501, USA; rjb2318@truman.edu; 3 Division of Plant Sciences, Southwest Research Center, University of Missouri, Columbia, MO 65211, USA; ThomasAL@missouri.edu

**Keywords:** American elderberry, total cyanogenic potential, cyanogenic glycosides, picrate method, solid phase extraction, UHPLC-MS/MS

## Abstract

Cyanogenic glycosides (CNGs) are naturally occurring plant molecules (nitrogenous plant secondary metabolites) which consist of an aglycone and a sugar moiety. Hydrogen cyanide (HCN) is released from these compounds following enzymatic hydrolysis causing potential toxicity issues. The presence of CNGs in American elderberry (AE) fruit, *Sambucus nigra* (subsp. *canadensis*), is uncertain. A sensitive, reproducible and robust LC-MS/MS method was developed and optimized for accurate identification and quantification of the intact glycoside. A complimentary picrate paper test method was modified to determine the total cyanogenic potential (TCP). TCP analysis was performed using a camera-phone and UV-Vis spectrophotometry. A method validation was conducted and the developed methods were successfully applied to the assessment of TCP and quantification of intact CNGs in different tissues of AE samples. Results showed no quantifiable trace of CNGs in commercial AE juice. Levels of CNGs found in various fruit tissues of AE cultivars studied ranged from between 0.12 and 6.38 µg/g. In pressed juice samples, the concentration range measured was 0.29–2.36 µg/mL and in seeds the levels were 0.12–2.38 µg/g. TCP was highest in the stems and green berries. Concentration levels in all tissues were generally low and at a level that poses no threat to consumers of fresh and processed AE products.

## 1. Introduction

American elderberry (AE), *Sambucus nigra* (subsp. *canadensis*) is a rapidly growing specialty crop in the United States [1]. Native to eastern and midwestern North America, AE is increasingly cultivated for its fruits and flowers that are used in a variety of foods, jellies, syrups, wines, and more importantly, dietary supplement products [2]. Elderberry is known for its nutritional and medicinal health benefits [3,4,5]. The fruit is rich in carbohydrates, fatty acids, organic acids, minerals, vitamins (A, B6 and C), essential oils, and is high in fiber [6,7]. Researchers have linked elderberry products to anti-inflammatory, anti-oxidant, anti-carcinogenic, anti-viral, anti-influenza, and antibacterial activities [3,8,9,10,11,12,13]. Whereas little scientific research has been conducted on AE as compared to its close relative, the European elderberry (EE), *Sambucus nigra* (subsp. *nigra*), both species are excellent sources of flavonoids, polyphenols and anthocyanins [3,8,9,14,15]. The elderberry industry is poised for major expansion and has increased significantly in sales (~0.8 to 107.6 M dollars) between 2011 and 2019 [16]. However, its competitiveness with other herbal dietary supplements [16] may by hampered in part due to uncertainty regarding the presence of cyanogenic glycosides (CNGs) and/or their putative toxicity.

**Figure 1:**
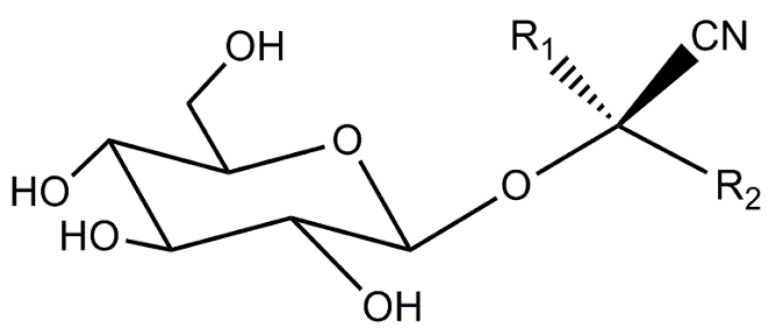
Generic structure for a cyanogenic glycoside, where R_1_ is often methyl or a proton and R_2_ is a variable organic group.

Cyanogenic glycosides are naturally occurring molecules in plants (nitrogenous secondary plant metabolites) which consist of aglycone and a sugar moiety [17,18]. A generalized structure is shown in Figure 1. There are about 60 CNGs widely distributed in the plant kingdom, occurring in over 2600 plant species representing more than 130 families [19,20,21,22]. CNGs are stored in vacuoles within plant cells, separating them from plant hydrolyzing endogenous enzymes (β-1,6-glycosidases and hydroxynitrile lyases) [17,23]. Although intact CNGs are nontoxic, endogenous plant enzymes can react with CNGs, and release hydrogen cyanide (HCN) causing potential toxicity issues [17,18,19,24,25]. Most CNG-containing plants also produce these endogenous enzymes so when their tissues are disrupted, for example by crushing, herbivory, or disease, CNGs can react with endogenous enzymes resulting in the release of HCN [23,26]. In plants, CNGs serve as important chemical defense compounds against herbivores and pathogens [19,21,27]. Clinical trials have shown mixed results regarding the potential of amygdalin (a CNG found in *Sambucus*) in cancer treatment and as a cough suppressant in various preparations [28,29]. In humans, consumption of cyanogenic plants can cause sub-acute cyanide poisoning (depending on dose) with symptoms including anxiety, headache, vomiting, nausea, abdominal cramps diarrhea, dizziness, weakness and mental confusion. Acute cyanide toxicity in humans (0.5–3.5 mg kg^−1^ body weight) [18,19] can result in decreased consciousness, hypotension, paralysis, coma and even death [17,18,19,24,25,30,31]. Acute cyanide poisoning has been reported from the ingestion of apricot kernels [32], bitter almonds [33], and cassava [34].

The only study previously made on *Sambucus canadensis* (American elderberry) is by Buhrmester et al. [20]. They examined the presence or absence of cyanogenic glycosides for individuals from nine populations of *Sambucus canadensis* L. (elderberry) in east-central Illinois. The study tested for cyanogenic glycosides in the leaves. Of the nine elderberry populations examined, only one population had a test positive for HCN production each of the three times tested. In another population the production of HCN was highly variable. The cyanogenic glycoside was determined to be (*S*)-sambunigrin by gas chromatographic separation of the TMS-derivative.

A review of the medical literature revealed no reports of elderberry juice poisoning in the past 30 years. The Centers for Disease Control and Prevention [35] did issue a bulletin about a poisoning incident on 26 August 1983 involving a group in California attributed to consumption of juice prepared from fresh wild elderberries along with leaves and stems (most likely blue elderberry, *Sambucus cerulea*) [35]. Cyanide was initially implicated in the incident, but was subsequently disproven. There remains uncertainty as to the presence of CNGs in elderberry juice and its products. Recent studies of European elderberry by Senica et al. [25,36] reported average levels of sambunigrin in fresh and processed berry products ranging between 0.8 and 18.8 µg/g [25] and higher amounts in elder leaves (27.68 and 209.61 µg/g FW) [36]. Koss-Mikolajczyk et al. [37] in similar work however recorded no quantifiable amounts of CNGs. To date, no exhaustive work has been completed on AE to conclusively ascertain the presence, forms, and levels of CNGs in ripe and unripe berries.

Traditional and modern food-processing techniques such as chopping, grinding, and heating are used to reduce the potential toxicity of plants containing CNGs [38,39,40]. However, the effectiveness of these techniques depends on the processing method [40], the plant tissue, and the intended processed forms. Soaking, for instance, may be effective when CNGs are soluble in the solution (discarded later) without enzymatic degradation [39,40,41]. Boiling can inhibit the activity of endogenous β-glucosidase due to high temperatures and halt the production of HCN [39]. However, this is only partially effective in reducing HCN because some CNGs are relatively heat stable [39] and HCN is water soluble [42]. Therefore, if CNGs do not hydrolyze due to enzyme inactivation, toxicity may still result from catabolism of these compounds in the gastrointestinal tract [43,44].

Quantification of CNGs can be made either indirectly (by determining HCN released after hydrolysis) or directly (by determining the intact glycoside) [17,19]. A very sensitive, reproducible and robust liquid chromatography/mass spectrometry-based method (LC-MS/MS) was developed and optimized for accurate identification and quantification of intact CNGs. Ultrahigh-performance liquid chromatography triple-quadrupole mass spectrometry (UHPLC-MS/MS) was used for this purpose [45,46]. A complimentary picrate paper method was modified to assess the total cyanogenic potential (TCP) by determining the total cyanide concentration following action of endogenous enzymes with CNGs. Analysis was performed using a camera-phone and UV-Vis spectrophotometry. In this study, we examine different elderberry fruit tissues. This study provides definitive and much needed answers to lingering questions regarding the safety of AE.

## 2. Results and Discussion

### 2.1. Picrate Paper Method

**Figure 2:**
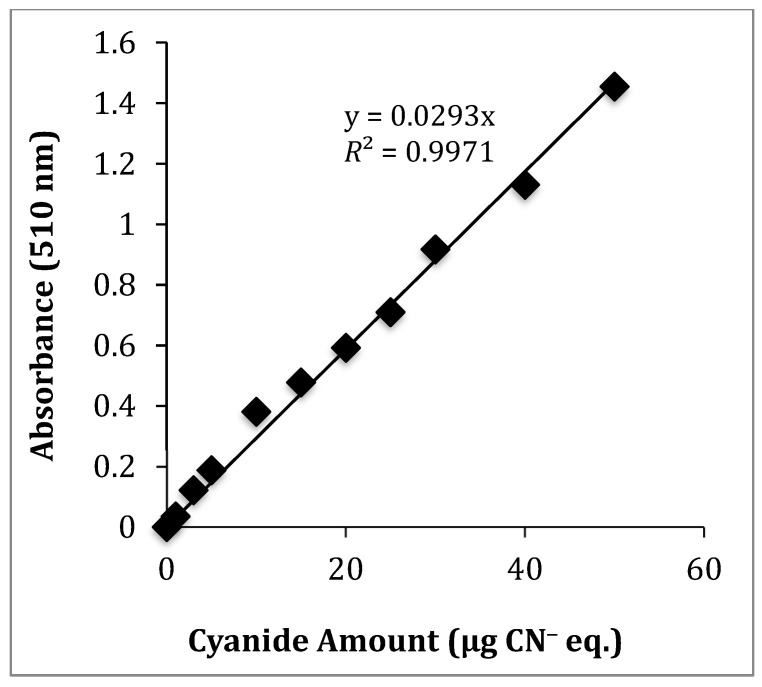
Calibration curve for the amount of CN^−^ eq. produced by reaction of picric acid with HCN using amygdalin as a CNG standard measured by UV-Vis spectrophotometry at λ_max_ = 510 nm.

The TCP was first determined by adapting a picrate paper method originally developed by Bradbury and co-workers [47]. This is a colorimetric method where picrate paper changes color in the presence of HCN. It is based on the reaction of picric acid with HCN. The method is described in more detail in Section 3.5. Amygdalin was used as a CNG standard to generate HCN. Cyanide equivalent (CN^−^ eq.) solutions were prepared from a 1000 µg/mL KCN/NaOH stock solution to develop a calibration curve over the range of 1 to 100 µg/mL. The observed color change of the picrate paper for amygdalin improved significantly and became consistent when the adapted method [47] was modified by using minimal liquid (<0.5 mL water). This was to enhance the HCN reaction with picric acid since HCN is highly water-soluble [42]. A standard calibration curve showing the amount of CN^−^ eq. with its corresponding absorbance is shown in Figure 2, using amygdalin as the cyanide source. Supplemental Figure S1 shows the corresponding standard curve using a camera-phone as a detector. Supplemental Figure S2 shows the expanded UV-Vis data from 0 to 10 μg. Table 1 summarizes the LLOD, upper limit of quantification (ULOQ), and the regression coefficients (*R*^2^-values) for camera-phone and UV-Vis analysis. UV-Vis showed better linearity compared to camera-phone method in repeated analysis.

**Figure 3:**
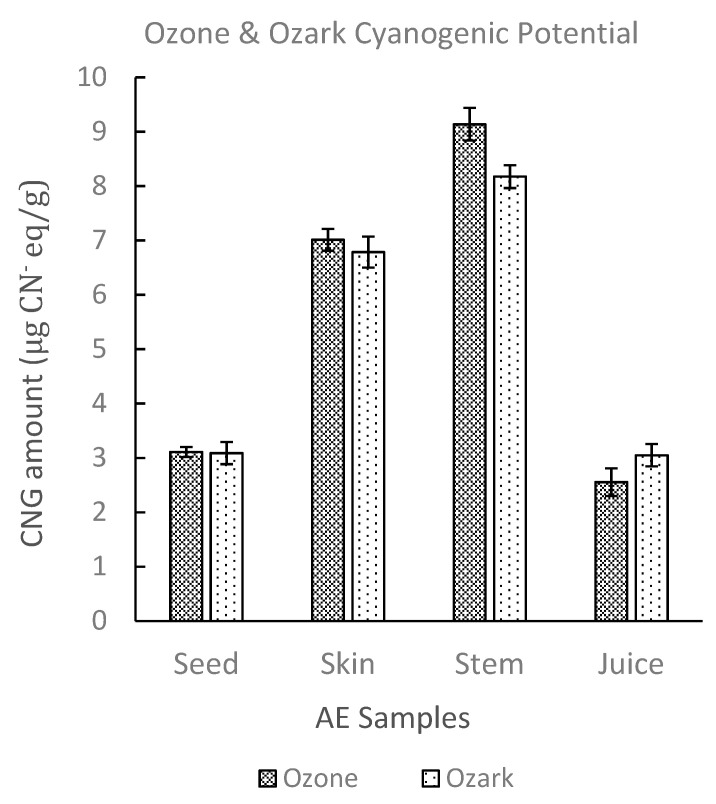
Total cyanogenic potential for different types of tissue of Ozone and Ozark AE genotypes. The amounts of CNGs in these genotypes were determined using UV-Vis spectrophotometry. The error bars represent the standard deviation of at least three replicate samples.

Qualitative inspection of the picrate paper strips showed no visible color change for the commercial elderberry juice sample. UV-Vis analysis of the picrate paper test strips detected no quantifiable amount of cyanide (<0.14 μg CN^−^ eq.). Two different AE genotypes, Ozark and Ozone, were then analyzed using the picrate paper method. Sample tissues (juice, skin, stem, seeds) for each genotype showed no visible color change on qualitative assessment (Supplemental Figure S3). Generally, results obtained for lyophilized samples were comparable to fresh samples. Quantitative determination by UV-Vis revealed low levels of cyanide with average amounts ranging from (2.60–9.20 µg CN^−^ eq.)/g of sample. TCP levels obtained were comparable for both AE genotypes and for all tissue types of Ozone and Ozark, respectively. TCP amounts increased in the order juice < seeds < skin < stem for both genotypes as shown in Figure 3 [48].

**Figure 4:**
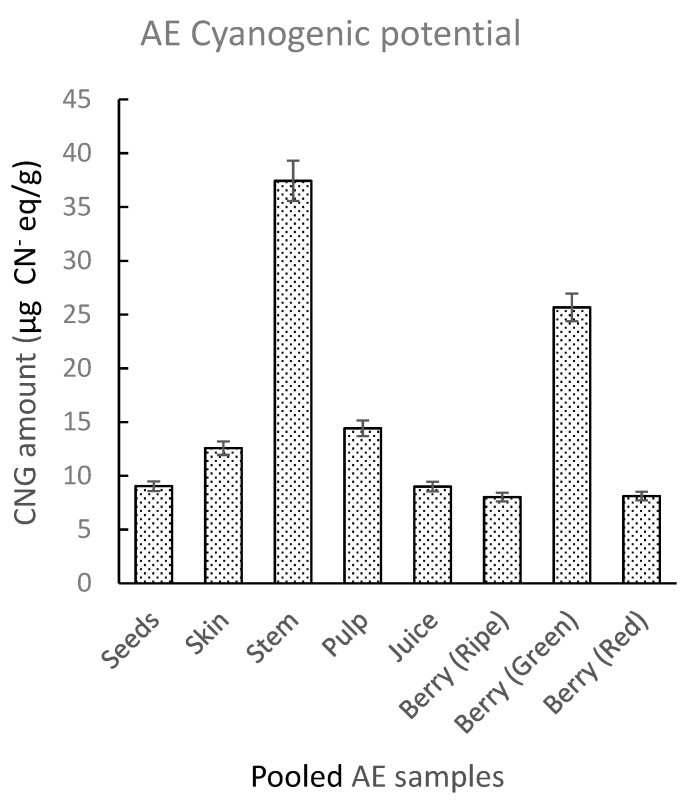
Total cyanogenic potential for different types of AE tissue of pooled samples made up of five different genotypes. The amounts of CNGs in pooled samples were determined using UV-Vis spectrophotometry. The error bars represent the standard deviation of at least three replicate samples.

A set of pooled AE samples was generated using five AE genotypes (Ozark, York, Wyldewood, Ocoee, and Bob Gordon). The pooled samples were divided into different types of tissue. These included seeds, skin, pulp, stems, juice, and whole green, red, and ripe berries. Qualitative inspection of picrate paper test strips for pooled AE samples showed a visible faint color change for the green berries and stems (Supplemental Figure S4). UV-Vis analysis showed the highest CN^−^ levels for stems and green berries with lower amounts for the other tissue types (Figure 4). TCP levels in analyzed tissues increased in the order: whole ripe berries < whole red berries < juice < seeds < skin < pulp < whole green berries < stem, with highest average amounts in the stems (37.43 ± 9.19 µg CN^−^ eq./g) and whole green berries (25.6 ± 5.07 µg CN^−^ eq./g). Koss-Mikolajczyk et al. [37] in a recent EE study observed a weak and unstable signal for a peak corresponding to sambunigrin which decreased with advancing stage of ripeness in elderberry fruit. In another study, Zahmanov et al. [49] reported metabolic differences in mature and immature fruits, and plant leaves of *Sambucus ebulus*. These observations may account for the slightly higher levels recorded in green berries. Although the CNG amounts in the stems and green berries are not sufficient to pose a threat of toxicity, it is nevertheless advisable to carefully exclude green elderberries and stems during juice preparation.

Two different types of seeds from Gala and Granny Smith apples were obtained and prepared for analysis as discussed in Section 3.5. Apple seeds were chosen as their TCP levels are known and should be readable using the picrate paper method. Color change on the picrate paper test strip for the apple seeds occurred swiftly at room temperatures even before test strips were transferred into the oven for overnight heating (30–40 °C). A deep red color change was observed on inspection for both fresh and lyophilized samples (Supplemental Figure S5). UV-Vis analysis showed high average cyanide amounts (TCP) ranging from (497.50–603.20 µg CN^−^ eq.)/g of apple seeds. TCP levels in analyzed seeds were higher in Granny Smith as compared to Gala apple varieties. These results were comparable to available literature [17,50].

Results from the endogenous enzymes test made using pooled AE stems and green berries revealed higher cyanide levels in samples with added β-glucosidase than those without added β-glucosidase (Supplemental Figure S6). Approximately 77% and 33% more cyanide were measured in pooled AE stems and whole green berries, respectively, with added β-glucosidase. These findings indicated that while AE contains endogenous β-glucosidase enzymes sufficient to initiate self-hydrolysis of CNGs, it may not be sufficient for complete hydrolysis of all CNGs (55–75%) when the tissues are disrupted. This implies that not all available CNGs in elderberry may necessarily be able to transform into HCN. These observations are supported in a similar analysis by Miller et al. [51] using foliage of the tropical trees *Beilschmiedia collina* and *Mischocarpus spp*. Apple seeds however showed no appreciable change in cyanide concentration with or without addition of β-glucosidase enzymes (Supplemental Figure S7), thus indicating that the seeds of the apple varieties used contain sufficient endogenous β-glucosidase for complete hydrolysis of all CNGs when the tissues are disrupted. The picrate paper test method is quick and simple and could serve as an effective field test for elderberry producers.

### 2.2. UHPLC MS/MS Method of Analysis

#### 2.2.1. Method Development and Optimization

**Figure 5:**
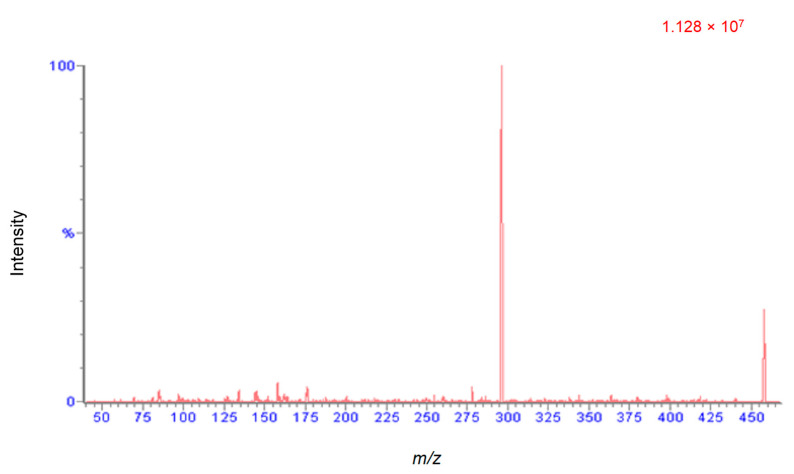
ESI positive mode product ion (296 *m*/*z*, product ion) spectrum for amygdalin (465 *m*/*z*, precursor ion).

**Figure 6:**
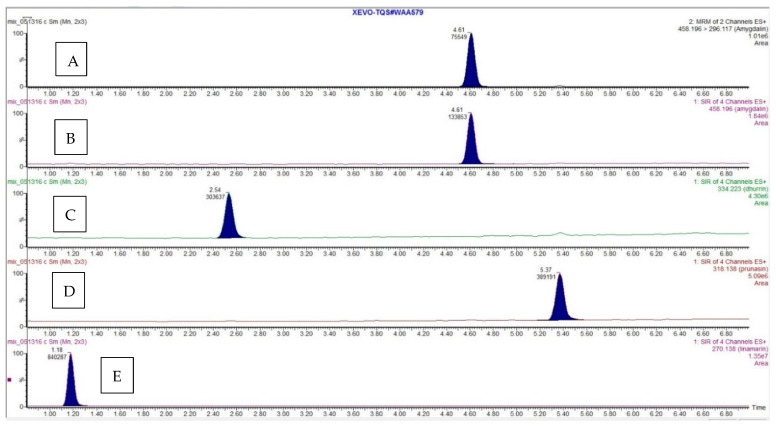
Ion chromatograms for (**A**) amygdalin (MRM), (**B**) amygdalin, (**C**) dhurrin, (**D**) prunasin, and (**E**) linamarin (SIR). Retention times in min. are: 4.61, 4.61, 2.54, 5.37, and 1.18, respectively.

An attempt was made to find multiple reaction monitoring (MRM) transitions for four cyanogenic standards (CNS) using both electrospray ionization (ESI) and atmospheric pressure chemical ionization (APCI) sources. Positive and negative ionization modes were performed for each standard with both ionization techniques. The only successful MRM transition identified was for amygdalin in ESI positive mode. Figure 5 shows the positive mode product ion (296 *m*/*z*, product) spectrum for amygdalin (465 *m*/*z*, precursor). All other standards readily formed sodium adducts, which did not sufficiently fragment due to their high stability. Alternative mass spectrometry scans were investigated to overcome this problem. Quantification for all four CNS’s were performed using selected ion recording (SIR) mode. The developed UHPLC and MS method displayed excellent separation of the four standards and exhibited retention time repeatability and good peak shape. A chromatogram for the separation with retention times (RT) and scanning modes is shown in Figure 6. It took less than 6 min to separate and elute all 4 CNS.

Standard calibration curves showed good linear correlations (*R*^2^ values) between integrated peak areas and known CNS concentrations. The lower limit of detection (LLOD) was determined based on a signal to noise ratio of three and a targeted coefficient of variation [52] (CV% ≤ 20%, for seven repeated injections) for confirmation. A linear range with lower (LLOQ, S/N =10, CV% ≤ 20%) and upper (ULOQ) limit of quantification was determined. The ULOQ was determined as the highest concentration of the linear curve beyond which the linearity breaks. Details are summarized in Table 2.

#### 2.2.2. Optimized Extraction, Recovery and Matrix Effect

Selecting the most appropriate extraction solvent was key to development of the extraction methodology. Recoveries from aqueous ethanol or methanol combinations were evaluated. Higher recoveries were obtained with 75% methanol extraction with overnight shaking (16–24 h) at room temperature and 30 min sonication at 30 °C as compared to other extraction methods and conditions. The recovery (RE) and matrix effect (ME) were evaluated by an approach based on responses from pre-extraction spike matrix (a), post-extraction spike matrix (b) and a neat spike standard (c). RE and ME were calculated using Equations (1) and (2) [53] (where +ME implies ion enhancement, −ME implies ion suppression). Table 3 below compares the mean recoveries and standard deviations for 30 min sonication at 30 °C to overnight shaking (16–24 h) at room temperature with intended spike concentration of 1000 ng/mL and 100 ng/mL CNS mixture. ME estimation was found to range between 10.20 and 18.34%. This was a negative estimation and as such indicated some degree of ion suppression. Although further dilution of sample matrix from 10 to 1000-fold reduced this value appreciably, it also decreased the sensitivity of sample detection. Hence a 10-fold dilution was used.

(1) $$\mathrm{RE}\;=\frac{a}{b}\times 100$$

(2) $$\mathrm{ME}\;=\;\left[\left(\frac{b}{c}\right)-1\right]\times 100$$

#### 2.2.3. Optimized SPE Method

**Figure 7:**
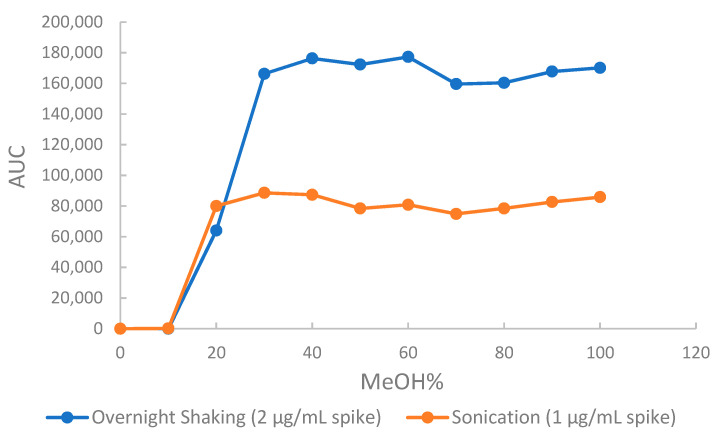
Elution profile for amygdalin as a function of methanol content in the extraction solvent. (AUC is the area under the curve).

To assess and evaluate the elution solvent strength in the SPE method, an elution profile showing methanol and water proportions from 0:100 to 100:0 (*v*/*v*) versus peak area was made for all four standards. Figure 7 shows the elution profile for amygdalin. Evaluation of these profile diagrams revealed 30–40% methanol as the best solvent strength for elution of all four CNS. Confirmation using water methanol proportions from 0:40 to 40:0 (*v*/*v*) was made to determine the best elution solvent as 35% methanol.

#### 2.2.4. Sample Test

**Figure 8:**
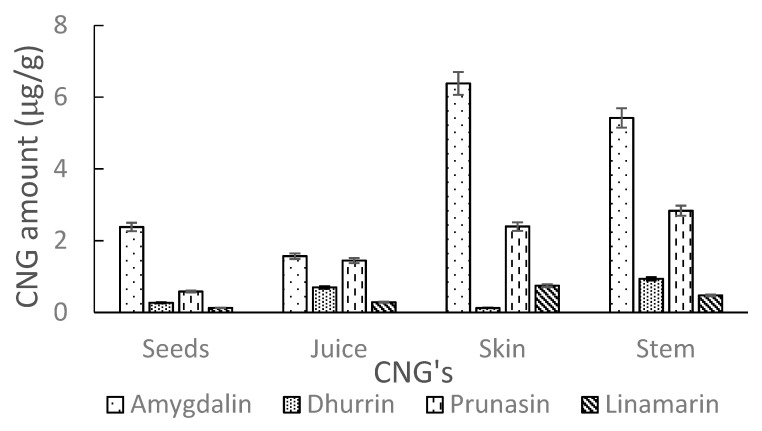
Amounts of CNGs (µg/g) in tissues (seeds, juice, skin and stem) of Ozone elderberry samples as measured by UHPLC-MS/MS.

**Figure 9:**
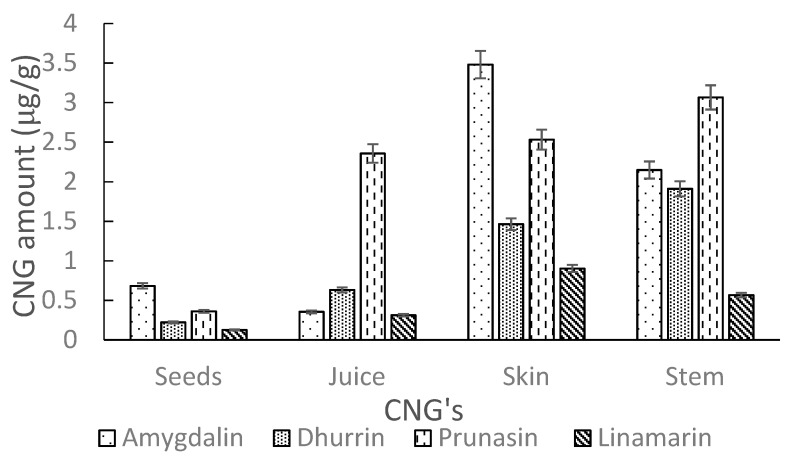
Amounts of CNGs (µg/g) in tissues (seeds, juice, skin and stem) of Ozark elderberry samples as measured by UHPLC-MS/MS.

The developed UHPLC-MS/MS method, as detailed in Section 3.6, was used to determine the levels of intact CNGs in different AE samples. Analysis of commercial elderberry juice showed no quantifiable amounts of CNGs. However, extracts of Ozark and Ozone elderberry tissues (seeds, juice, skin and stem) for both lyophilized and fresh samples showed low traces of CNGs (amygdalin, dhurrin, linamarin and prunasin). Levels of CNGs detected in lyophilized samples were comparable to fresh samples. The amounts (µg/g) in tissues were generally higher in Ozone compared to Ozark. Higher levels (µg/g) were recorded in the stems and skin tissues as compared to levels in the seeds and juice, respectively for the Ozone and Ozark samples. A detailed summary of amounts for each detected CNGs in AE tissues are shown in Table 4. Figure 8 and Figure 9 show the amounts of these cyanogens in tissues for Ozone and Ozark, respectively. The levels (µg/g) of CNGs in analyzed tissues increased in the order: linamarin < dhurrin < prunasin < amygdalin, respectively, for Ozone and Ozark samples tissues. In contrast to this trend, prunasin levels were highest in the juice and stems of Ozark AE.

In our UHPLC MS/MS, we are not able to distinguish between the two diastereomers, (*R*)-prunasin and (*S*)-sambunigrin. The two compounds were not uniquely separated by UHPLC using a C18 column. Further, their fragmentation patterns are very similar. Therefore, the prunasin concentrations should be viewed as a sum of the prunasin and sambunigrin concentrations.

A review of literature in similar areas of study found comparable results, but also revealed an interesting trend of observation. A recent study by Senica et al. [40] on the EE (subsp. *nigra*) reported average levels of sambunigrin (µg/g) in fresh berries (18.8 ± 4.3), processed juice (10.6 ± 0.7), tea (3.8 ± 1.7), spread (0.8 ± 0.19) and liqueur (0.8 ± 0.21). Our measured levels of (prunasin + sambunigrin) for AE are lower for fresh berries compared to EE. Senica et al. [36] in a similar work reported highest amounts of sambunigrin in elder leaves (27.68–209.61 µg/g FW), lower amounts in flowers (1.23–18.88 µg/g FW) and lowest amounts in berries (0.08–0.77 µg/g FW). In the work by Buhrmester et al. [20], also observed similar sambunigrin concentrations for AE leaves. Senica and co-workers concluded that the content of sambunigrin in elderberry changes depending on the growing altitudes (higher content on hill tops and lower in foothills) [36]. Another study by Koss-Mikolajczyk et al. [37] on EE (subsp. *nigra*) observed the highest signal for a peak detected as sambunigrin in the elder leaves although this peak became undetectable after one day of cold storage of extracts. It was also reported that the level of cyanogens in cassava leaves are 10 times more than in the roots [39]. Deductions from this trend of results suggest that the leaves of most cyanogenic plants may accumulate larger amounts of CNGs, with elder as no exception. The trend of results also corroborates the fact that elderberry juice, being it, AE or EE showed very low levels of CNGs.

The levels of CNGs detected in all tissues of AE samples were extremely low compared to levels of amygdalin detected in apple seeds (950–3910) µg/g, pressed apple juice (10–39) µg/mL and commercially available apple juice (1–7) µg/mL for 15 apple varieties [17]. Acute CN toxicity occurs at a concentration of 0.5–3.5 mg/kg of body weight. For cyanide in blood, the toxicity threshold for cyanide alone ranges from 0.5 to 1.0 mg/L, and the lethal threshold ranges from 2.5 to 3.0 mg/L [54]. Despite the high cyanide levels in apple seeds as revealed in the control picrate test (497.50–603.20 µg CN^−^ eq./g), signs of cyanide toxicity may occur in an average adult male of weight 82 kg, only after consuming about 14 or more apples including mastication of all associated seeds. This estimation was made considering the threshold value of 0.50 mg/kg body weight for cyanide toxicity, average TCP per seed of 550 µg/g (or 0.55 mg/g), the average weight of an apple seed (0.75 g), the average number of seeds per apple (7 or 8), and assuming maximum enzyme activity.

Different processing techniques such as chopping, grinding, soaking, fermentation, drying, roasting, boiling, and steaming have been used to remove or reduce the potential toxicity of cyanogens in plants [38,39,40]. The effectiveness of these processes is dependent on the specific processing method [40], the plant tissues and the intended processed forms. Boiling of juice for instance may have a different effect compared with boiling or soaking cassava chips where the associated water can easily be discarded [39,40,41]. This may be due to enzyme inactivation and solubilization of CNGs in discarded water [39]. A study by Montagnac et al. further indicated that the effectiveness of these techniques depends on the processing steps, the sequence utilized, and is often time-dependent [39]. They proposed that to increase the efficiency of cyanogen removal from cassava, efficient processing techniques should be combined [39]. For example, soaking, fermenting and roasting removes about 98% of cyanogens [39]. A recent study by Senica et al. [40] also showed that thermal processing, time and type of extraction solution greatly affected phenolics and cyanogenic glycosides in different elderberry products. They showed that higher processing temperatures decreased the levels of cyanogenic glycosides by 44% in elderberry juice, 80% in tea and as much as 96% in elderberry liqueur and spread [40]. It has been confirmed that pasteurization effectively decreases the levels of harmful compounds, such as cyanogenic glycosides [17,40]. Bolarinwa et al. [17] moreover observed that holding apple juice at room temperature for 120 min either before or after pasteurizing decreased the amygdalin content by about 19% compared to the original juice. These methods are very effective and can be applied to remove or further reduce the levels of CNGs in elderberry product. It is however important to establish that the types and levels of CNGs observed in AE are very low and pose no threat to consumers in the use of fresh or processed AE products.

## 3. Materials and Methods

### 3.1. Chemicals and Reagents

Water, acetonitrile (ACN), methanol, ethanol and formic acid were purchased from Fisher Scientific (Fair Lawn, NJ, USA, HPLC grade). β-glucosidase enzymes (250 mg lyophilized powder ≥ 6U/mg), amygdalin (1 g, ≥99%), dhurrin (1 mg, ≥98%), prunasin (1 mg, ≥95%) and linamarin standards (1 mg, ≥95%), together with picric acid (100 g moisten with water ≥ 98%) and potassium cyanide (≥98%) were purchased from Sigma Aldrich Chemical Co. (St. Louis, MO, USA). Whatman no.1 filter paper, sodium carbonate, sodium hydroxide and pH 8 phosphate buffer (500 mL) were also purchased from Fisher Scientific (Fair Lawn, NJ, USA). Plastic backing and hobby glue (adhesive neutral pH) were purchased from the Mizzou Store (Columbia, MO, USA).

### 3.2. American Elderberry Samples

Plant Material. Elderberry fruit samples were harvested from experimental field plots that were previously described in detail [2,45]. Briefly, a replicated evaluation of eight American elderberry genotypes was established in Missouri (USA) in 2008. Fruits from six genotypes (Bob Gordon, Ocoee, Ozark, Ozone, Wyldewood, and York) were harvested from one of the study sites (Mt. Vernon, MO, USA) at peak ripeness in August 2016, and promptly frozen. Frozen, de-stemmed, whole berries (>400 g) from the five genotypes were provided to the laboratory, along with frozen unripe and almost-ripe berries (green and red-colored, respectively). Additionally, hundreds of individual berries from each genotype were thawed and painstakingly separated into skins (epicarp), pulp (mesocarp), seeds, juice, and small green stems (pedicels) that connect the berry to the infructescence. After dissection, samples were re-frozen. For detailed CNG analysis, tissue and juice samples from the genotypes Ozone and Ozark were analyzed separately. Further, tissue and juice from five genotypes (Bob Gordon, Ocoee, Ozark, Wyldewood, and York) were combined into pooled samples for a broader analysis. Sufficient material was dissected to produce samples exceeding 10 mg.

Commercially processed elderberry juice was purchased from River Hills Harvest, Hartsburg, MO, USA.

### 3.3. Sample Preparation and Extraction

Berries were transferred into small zippered plastic bags, thawed, and gently pressed. The juice was transferred to a clean vial. Seeds were separated from skin and placed into different vials. 100 g of berries produced about 60 g of juice, 20 g of seeds, 12 g of skin, and some left-over stems. Between 5–10 g of each sample tissue (excluding juice) was transferred into 15 mL Eppendorf tubes, flash frozen for about 5 min in liquid nitrogen and freeze-dried for 24 h using a Labconco FreeZone 4.5 Liter Benchtop Lyophilizer (Labconco Corp., Kansas City, MO, USA) at −105 °C. The lyophilized samples were ground using a clean mortar and pestle to obtain about 3–5 g of homogenized seeds, stem and skin samples for extraction.

**Figure 10:**
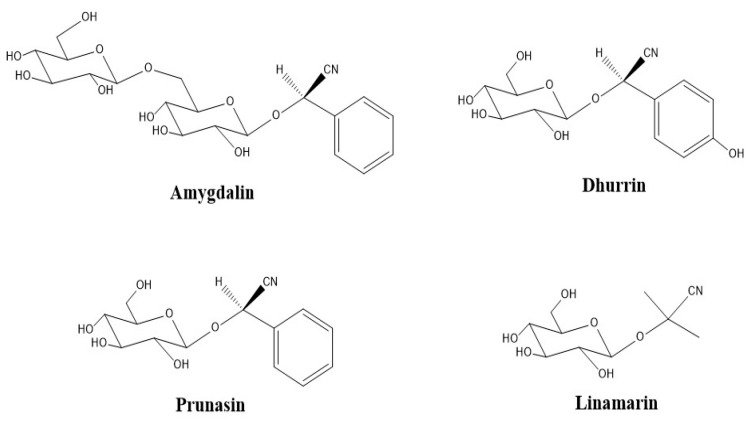
Cyanogenic glycosides standards used in this study: amygdalin, dhurrin, prunasin, and linamarin.

To obtain an optimized sample pretreatment and extraction, equal volumes of commercially processed elderberry juice, in replicates of 4, were spiked with varying amounts of 10 µg/mL cyanogenic standard (CNS) stock mixture (amygdalin, dhurrin, prunasin and linamarin, Figure 10) to obtain intended spike concentrations of 1000, 100, and 10 ng/mL. The solutions were extracted with 1 mL of different ethanol/methanol and water proportions from 60:40 to 80:20 (*v*/*v*). Extraction was performed via sonication (10–60 min) at 30 °C, overnight shaking (16–24 h) and 2 min vortexing at room temperature on a Genie 2 Shaker Mixer (Scientific Industries, Inc., Bohemia, NY, USA) at 600 rpm. Extracts were centrifuged for 15 min, dried under nitrogen gas and reconstituted in 1 mL of mobile phase (0.1% FA in ACN) for SPE clean up. Sample extraction was performed with both fresh and lyophilized samples.

### 3.4. Solid Phase Extraction (SPE)

An SPE method [24] previously used for the determination of amygdalin in almonds was adapted and optimized for our use. A Supelco Visiprep™ SPE vacuum manifold (Sigma-Aldrich, St. Louis, MO, USA) was used for this purpose. A vacuum of 10 in Hg (35 kPa) and a flowrate of about 1–2 drops/s was maintained throughout the process. A SPE cartridge (Sep-Pak Vac 3 cc (500 mg) C18 cartridge, Waters, Milford, MA, USA) was conditioned with 2 mL of methanol and equilibrated with 2 mL of water. 1 mL of the sample was loaded onto the column. An additional 1 mL of 0.1% FA in water was used to remove remaining residue in the extraction tube. The column was flushed with 2 mL of 0.1% FA in water. CNGs were finally eluted with 2 mL of varying proportions of methanol/water (0, 10, 20, 30 to 100% *v*/*v*). The extracts were dried under nitrogen gas, reconstituted into 0.1% FA in water and filtered through a 0.45 μm filter prior to UHPLC-MS/MS analysis.

### 3.5. Picrate Paper Method of Analysis

The picrate paper method is based on the reaction of enzymes that catalyze the release of HCN gas, which reacts with picric acid on a paper test strip producing 2,6-dinitro-5-hydroxy-4-hydroxylamino-1,3 dicyclobenzene inducing a color change (Supplemental Figure S8) [46].

A previously published picrate method described by Bradbury et al. [47] for the determination of the total cyanogenic content in cassava roots was adapted and modified for use. Briefly, the picrate paper was prepared beforehand by dipping a sheet of Whatman 3 MM filter paper in a picrate solution (1.4% *w*/*v* moist picric acid diluted in 2.5% *w*/*v* Na_2_CO_3_ solution), allowing the paper to air dry and cutting it into 3 cm × 1 cm strips. The strips were attached using a drop of PVA hobby glue to 5 cm × 1.2 cm clear plastic strips to keep the paper clear of the liquid. They were stored at 4 °C prior to use. Cyanide equivalent (CN^−^ eq.) solutions were prepared from a 1000 µg/mL KCN stock solution. The stock solution was prepared by dissolving KCN in 0.1M NaOH as the solvent. The calibration curve covered the range of 1 to 100 µg CN^−^ eq. and the method was verified using amygdalin as a positive control. One of the most complicated portions of this analysis is the enzymatic hydrolysis of amygdalin. Enzymes are macromolecular biological catalysts whose amount for a specific enzymatic activity is measured in Units (U). One U is defined as the amount of enzyme needed to catalyze the conversion of 1 micromole of substrate per minute [48]. Enzymatic degradation of amygdalin was achieved by adding 50 µL of 3U/mL β-glucosidase.

The commercially processed elderberry juice was tested for TCP along with the AE samples. 100 µL/100 mg each of lyophilized and fresh tissues of Ozone, Ozark and pooled AE samples were measured/weighed into clean 20 mL scintillation vials. 50 µL of β-glucosidase solution (3U/mL) in pH 8 phosphate buffer was added alongside the picrate paper and the vial was immediately closed with a screw stopper. Each sample analysis was made in replicates of four. Similar set-ups were made for amygdalin standards and blank (no amygdalin added). These were left overnight (16–24 h) in an oven at 30–40 °C.

Two control experiments were performed. The first was to confirm the effectiveness of the picrate paper test method to known literature. Seeds from two apple varieties, Granny Smith (GS) and Gala (G), were prepared and tested for TCP using the same protocol for the AE samples. The second control experiment used seeds (ground) from the two apple varieties and stems and green berries from AE pooled samples. This was to test for the presence of endogenous enzymes in the samples to assess the extent of self-hydrolysis of CNGs. To accomplish this, two different picrate paper set-ups were made, a control (without an external β-glucosidase solution) and a second typical picrate set-up (with an external β-glucosidase solution). Four replicates for each set-up were performed.

**Figure 11:**
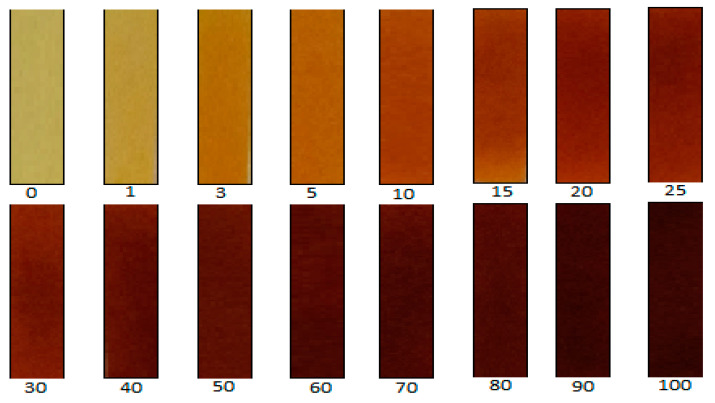
A picrate-paper cyanide color chart for qualitative analysis of CNGs for the range of 0–100 µg CN^−^ eq.

A simple and quick method of analyzing the reacted picric acid is by qualitative inspection. This appears to be an effective method for quantifying CN^−^ equivalents ranging between 1 and 100 µg. However, as the amount of CN^−^ eq. increases, the ability to differentiate between the colors decreases. A color chart shown in Figure 11 can be used to relate the color change of the paper to total amount of CN^−^ evolved. A semi-quantitative approach using a camera-phone as a detector was used. An image of a concentration from Figure 11 was converted from color to greyscale. This was done using Image J software (https://imagej.nih.gov/ij/index.html (accessed on 22 December 2020), version 1.46r, National Institutes of Health, Bethesda, MD, USA). The method generated mean intensity values corresponding to each CN^−^ eq. and was used to generate a calibration curve. Quantification was confirmed using a UV-Vis spectrometer (Agilent 8454 photodiode array, Agilent Technologies, Santa Clara, CA, USA). The reacted picrate paper test strip was extracted in 3.5 mL of water in cuvettes and the resulting solution analyzed at a wavelength (λ_max_) of 510 nm after standing for 30 min.

### 3.6. UHPLC-MS/MS Method of Analysis

Separation and analysis of cyanogenic glycosides were performed with a C18 column (Acquity BEH, 1.7 μm, 50 × 2.1 mm, Waters, Milford, MA, USA) using a Waters Acquity UHPLC coupled to a Xevo TQ-S triple quadrupole mass spectrometer (UHPLC-MS/MS). A previously published gradient [24] for the quantification of amygdalin in almonds was reduced from 20 min down to 10 min. The mobile phase included 0.1% formic acid in water (mobile phase A) and 0.1% formic acid in acetonitrile (mobile phase B). The gradient used was 95% A, 0−1 min; 95−80% A, 1−3 min; 80−40% A, 3−7 min; 40% B, 7−8 min and 95% A 8.1−10 min re-equilibration. The flow rate was 200 μL min^−1^ and the following conditions were used for the electrospray ionization (ESI) source: source temperature 150 °C, desolvation temperature 350 °C, capillary voltage 3.07 kV, cone voltage 21, and nebulizer gas 500 L h^−1^ N_2_. Argon was used as the collision gas. The collision energies were optimized and ranged from 17 to 30 eV for individual analytes. The column and sample temperatures were 40 ° and 10 ° C, respectively. The ESI source was operated in the positive ion mode. Instrument control and data processing were performed by using MassLynx software (version 4.1, Waters, Milford, MA, USA). Cyanogenic standard solutions were prepared with concentrations ranging from 1 ng mL^−1^ to 10 μg mL^−1^. All analyses were done in triplicate along with a blank. The interday and intraday precisions of the method had a CV% of less than 5%.

### 3.7. Statistical Analysis

For the determination of cyanide by UV-Vis and cyanogenic glycosides by LC-MS/MS, samples were prepared in three biological and three analytical replicates for each sample. Statistical analyses were performed in Excel (Microsoft Office 2016). Results are expressed as the mean ± standard error of mean (SEM). Additionally, the coefficient of variation for six (6) repeated injections (CV% ≤ 20%) was used to confirm candidate concentrations for LLOQ and LLOD.

## 4. Conclusions

The UHPLC-MS/MS and picrate paper methods developed were used to reliably determine the intact CNGs and assess the TCP in various AE fruit tissue. No quantifiable trace of cyanide or CNG was detected in commercial elderberry juice. Moreover, traces of CNGs (amygdalin, dhurrin, (prunasin + sambunigrin), and linamarin) detected in tissues of AE samples were generally low with lower levels in the juice and seeds as compared to stems and skin. TCP assessed in both pure and pooled AE sample tissues were generally low with higher concentrations recorded in pooled stems and unripe (green) berries. The picrate paper method can also be used to help detect the presence of CNGs. A camera-phone and UV-Vis spectrophotometer can both be used as a detector. The camera-phone can give results easily with limits of detection that are useful for CNG analysis. Although the TCP and CNGs levels in tissues of AE pose no threat to consumers, it is advisable to separate out the stems, green berries and leaves [36] from AE ripe berries during product preparation.

## Figures and Tables

**Table 1:** LLOD, ULOQ, and Pearson coefficients (*R*^2^-values) for calibration curves from camera-phone and UV-Vis analysis using amygdalin as a CNG standard.

Method/µg CN^−^ eq.	ULOQ	LLOQ	*R* ^2^
UV-Vis	50	0.14	0.9971
Camera-phone	50	1.59	0.9889

**Table 2:** Summary of Pearson coefficient, detection and quantification limits (ng/mL) for CNGs.

Parameters ng/mL	MRM Amygdalin	SIR			
		Amygdalin	Dhurrin	Prunasin	Linamarin
**LLOD**	0.3	3	3	3	1
**LLOQ**	1	10	10	5	5
**ULOQ**	8000	8000	6000	6000	2000
**R** ^2^	0.9998	0.9998	0.9983	0.9984	0.9910

**Table 3:** Mean recoveries and standard deviations for spike concentrations of 100 and 1000 ng/mL.

CNG Standards/	Conc.	MRM		SIR							
Mean Recovery%	(ng/mL)	Amygdalin		Amygdalin		Dhurrin		Prunasin		Linamarin	
		Mean RE%	SD%	Mean RE%	SD%	Mean RE%	SD%	Mean RE%	SD%	Mean RE%	SD%
Sonication	1000	85.40	1.01	86.14	1.61	95.34	2.1	91.35	0.95	95.26	1.98
(30 min at 30 °C)	100	91.43	0.92	92.14	3.11	88.54	2.04	79.14	5.1	92.1	0.96
Overnight shaking	1000	93.69	0.31	91.40	0.98	98.19	0.73	106.98	0.86	112.21	0.62
(16–24 h)	100	81.70	3.61	101.54	2.98	109.99	2.75	87.94	1.85	98.11	2.13

**Table 4:** Amounts (µg/g) of CNGs found in tissues of Ozone and Ozark AE samples.

Elderberry Samples		Concentration ± Standard Deviation (µg/g)			
		Amygdalin	Dhurrin	Prunasin	Linamarin
**Seeds**	Ozone	2.38 ± 0.09	0.27 ± 0.05	0.58 ± 0.04	0.12 ± 0.06
	Ozark	0.68 ± 0.12	0.22 ± 0.03	0.36 ± 0.05	0.13 ± 0.05
**Juice**	Ozone	1.57 ± 0.08	0.70 ± 0.12	1.45 ± 0.06	0.29 ± 0.03
	Ozark	0.36 ± 0.03	0.63 ± 0.04	2.36 ± 0.08	0.31 ± 0.01
**Skin**	Ozone	6.38 ± 0.40	0.12 ± 0.08	2.39 ± 0.04	0.75 ± 0.06
	Ozark	3.48 ± 0.14	1.46 ± 0.20	2.53 ± 0.08	0.90 ± 0.11
**Stem**	Ozone	5.42 ± 0.12	0.94 ± 0.06	2.84 ± 0.02	0.48 ± 0.04
	Ozark	2.15 ± 0.17	1.91 ± 0.03	3.07 ± 0.06	0.57 ± 0.06

## Data Availability

Original data is available upon request to the corresponding author.

## Supplementary Materials

The following are available online: Figure S1: Picrate-paper results for the using a camera-phone to detect the presence of CN^−^ using amygdalin. Figure S2: Picrate-paper results for the using a UV-Vis spectrophotometer in the low concentration range (0–10 μg) to detect the presence of CN^−^ using amygdalin. Figure S3: Picrate Paper results for tissues of Ozone and Ozark AE samples, Figure S4: Picrate paper results for pooled AE samples, Figure S5: Picrate paper results for apple seeds and juice, Figure S6: Picrate paper results for endogenous enzymes test for pooled AE tissues, Figure S7: Picrate paper results for endogenous enzymes test for fresh and lyophilized apple seeds, Figure S8: Cyanide reaction with picric acid.

## Sample Availability

Not available.

## Abbreviations

ACN	acetonitrile
AE	American elderberry
APCI	atmospheric pressure chemical ionization
CDC	Centers for Disease Control and Prevention
CNGs	cyanogenic glycosides
CNS	cyanogenic standards
EE	European elderberry
ESI	electrospray ionization
FA	formic acid
HCN	hydrogen cyanide
HPLC-DAD	High-performance liquid chromatography with photo diode array detectors
LLOQ	lower limit of quantification
LLOD	lower limit of detection
ME	matrix effect
MRM	multiple reaction monitoring
RE	recovery
RT	retention time
SD	standard deviation
SIR	selected ion recording
S/N	signal to noise ration
SPE	solid-phase extraction
TCP	total cyanogenic potential
UHPLC-QqQ-MS/MS	Ultra-high performance liquid chromatography triple-quadrupole mass spectrometry
ULOQ	upper limit of quantification
UV-Vis	ultraviolet visible spectrophotometry
