# Supplementary material for: Cyanogenic Glycoside Analysis in American Elderberry
Source: Molecules. 2021 Mar 4;26(5):1384. doi: 10.3390/molecules26051384 (PMC7961730; doi:10.3390/molecules26051384)
Supplement: Supplementary file 1 [file molecules-26-01384-s001.pdf]

Supplementary Materials

Cyanogenic glycoside analysis in American elderberry

Michael K. Appenteng [1], Ritter Krueger [1], Mitch C. Johnson [1], Harrison Ingold [1], Richard Bell [3], Andrew L. Thomas [2] and C. Michael Greenlief [1,*]

[1] Department of Chemistry, University of Missouri, Columbia, MO 65211, USA; mkappenteng@mail.missouri.edu (M.K.A.); rmkqm2@mail.umkc.edu (R.K.); mcjohnson0989@gmail.com (M.C.J.); hgidn9@mail.missouri.edu (H.I.)
[2] Department of Chemistry, Truman State University, Kirksville, MO, USA; rjb2318@truman.edu
[3] Division of Plant Sciences, Southwest Research Center, University of Missouri, Columbia, MO 65211, USA; ThomasAL@missouri.edu
* Correspondence: greenliefm@missouri.edu; Tel: +01-573-882-3288; Fax: 573-882-2754

1. **Figure S1:** Picrate-paper results for the using a camera-phone to detect the presence of CN^- using amygdalin.
2. **Figure S2:** Picrate-paper results for the using a UV-Vis spectrophotometer in the low concentration range (0–10 μg) to detect the presence of CN^- using amygdalin.
3. **Figure S3**: Picrate-paper results for tissues of Ozone and Ozark AE samples.
4. **Figure S4**: Picrate-paper results for pooled AE samples
5. **Figure S5:** Picrate-paper results for apple seeds and juice
6. **Figure S6:** Picrate-paper results for endogenous enzymes test for pooled AE tissues.
7. **Figure S7:** Picrate-paper results for endogenous enzymes test for raw and lyophilized apple seeds.
8. **Figure S8**: Cyanide reaction with picric acid.

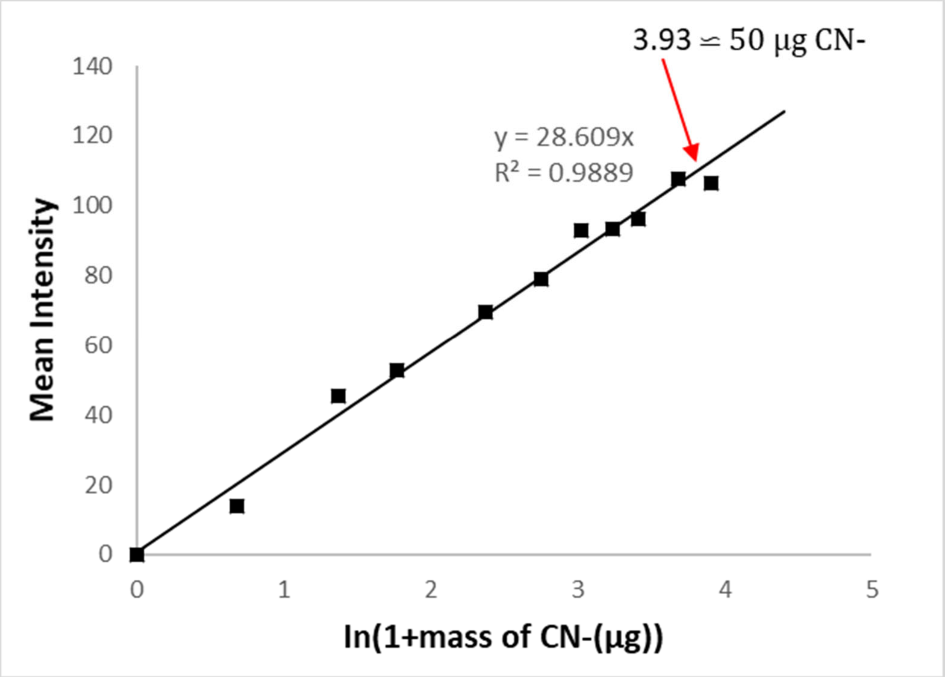

Figure S1. Calibration curve for CN^- eq. using amygdalin as the standard by the picrate-paper method and a camera-phone as the detector. The curve generated from a plot of mean intensity values verses the natural logarithm of cyanide equivalent amounts. An image the picrate-paper was converted from color to greyscale. Conversion to greyscale helped increase the upper limit of detection where color saturation became an issue. The conversion was done using Image J

software (https://imagej.nih.gov/ij/index.html). The mean intensity values corresponding for each CN⁻ eq. were used to generate the calibration curve.

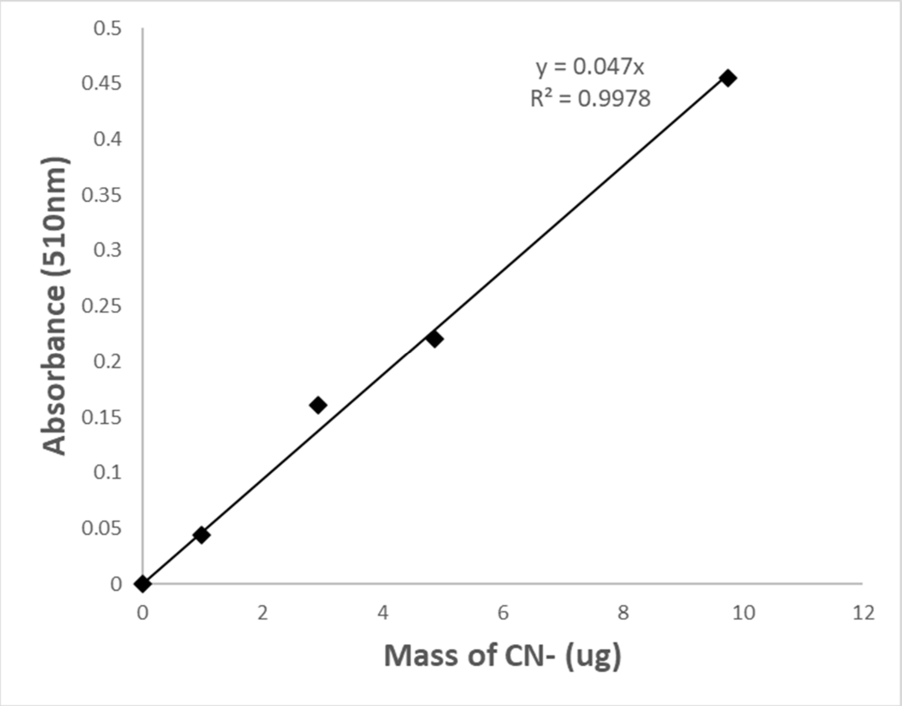

Figure S2. Calibration curve for CN^- eq. using amygdalin as the standard by the picrate-paper method and using a UV-Vis spectrophotometer as the detector (λ_{max} = 510 nm). The curve shows as expanded region at the lowest concentrations (0 to 10 μg). The curve generated from a plot of average absorbance verses cyanide equivalent amounts.

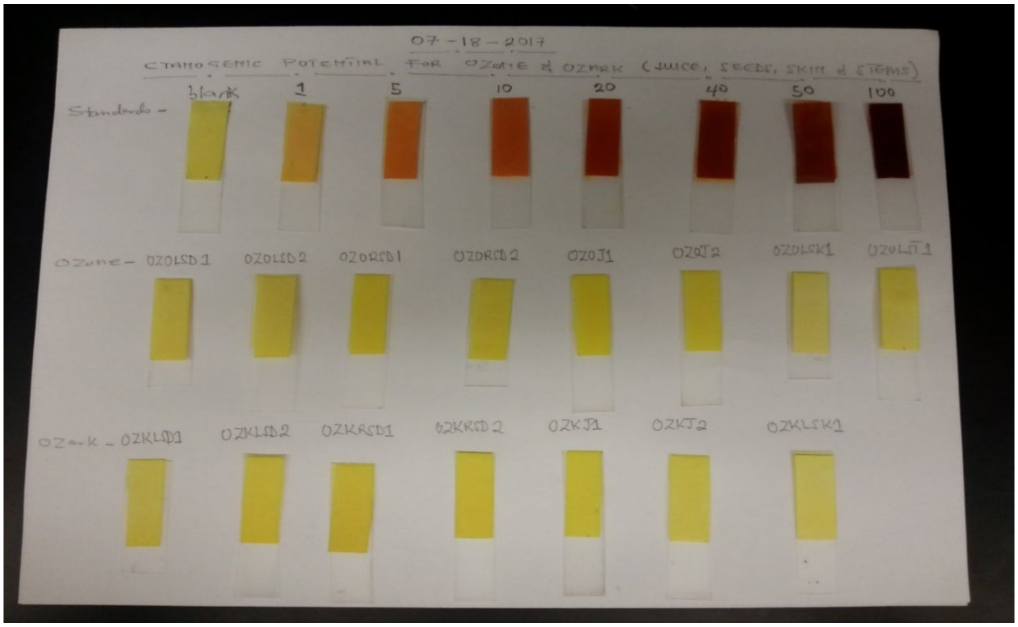

Figure S3. Picrate-paper results for different tissues (juice, seeds, stems, and skin) of Ozone and Ozark AE samples. The top row of strips indicates the color changes for different concentrations of amygdalin (μg) as CN^- standard. The units of concentration for the CN^- standards is in μg. The middle row from left to right are Ozone AE samples: OZOLSD1 and OZOLSD2 are lyophilized seeds, OZORS1 and OZORS2 are raw/fresh seeds, OZOJ1 and OZOJ2 are fresh juice, OZOLSK1 is lyophilized skin, and OZOLST1 is lyophilized stems. The bottom row from left to right are Ozark AE samples: OZKLSD1

and OZKLSD2 are lyophilized seeds, OZKRSD1 and OZKRSD2 are raw/fresh seeds, OZKJ1 and OZKJ2 are fresh juice, and OZKLSK1 is lyophilized skin.

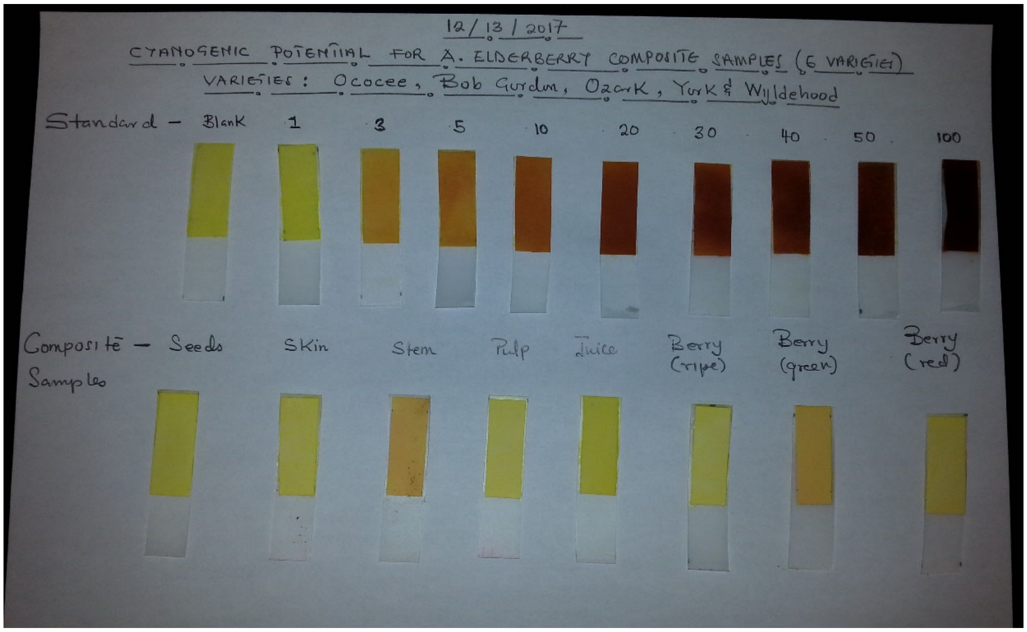

Figure S4. Picrate-paper results for different tissues (juice, seeds, stems, and skin) of the pooled AE samples. Elderberry pulp, fresh green, red and ripe berries are also analyzed. The pooled samples are a mixture of different AE genotypes. The top row of strips indicates the color changes for different concentrations of amygdalin (µg) as CN^- standard. The units of concentration for the CN^- standards is in µg.

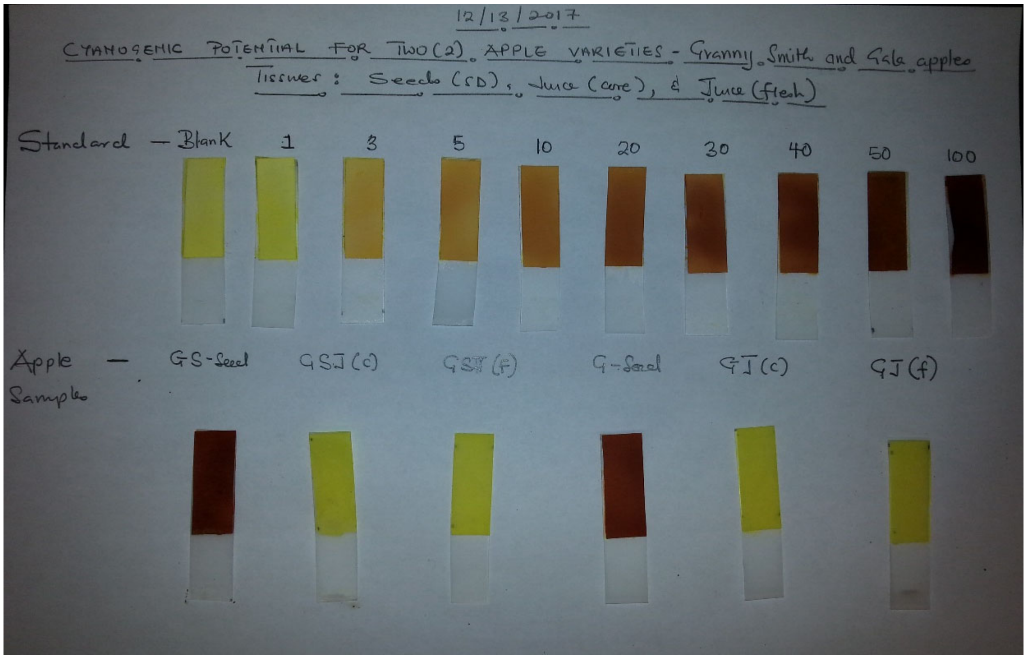

Figure S5. Picrate-paper results for results for apple seeds, core, flesh, and juice. Granny Smith (GS) and Galas (G) apples were used in the test. The top row of strips indicates the color changes for different concentrations of amygdalin (µg) as CN^- standard. The units of concentration for the CN^- standards is in µg. The bottom row shows picrate-paper results for apple samples. From left to right the samples are Granny Smith seeds (GS-seed), Granny Smith juice taken near the core (GSJ(c)), Granny Smith juice taken from the main part of the apple (GSJ(f)), Gala seeds (G-seed), Gala juice taken near the core (GJ(c)), and Gala juice taken from the main part of the apple (GJ(f)).

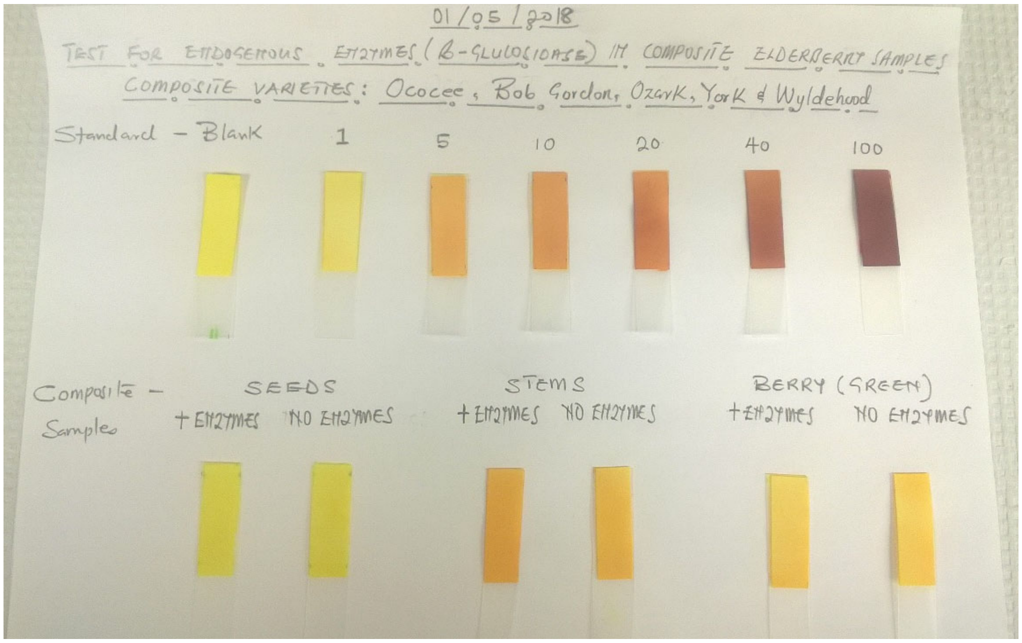

Figure S6. Picrate-paper results for different tissues (berries, seeds, and stems) of the pooled AE samples with and without the addition of endogenous enzymes. The pooled samples are a mixture of different AE genotypes. The top row of strips indicates the color changes for different concentrations of amygdalin (µg) as CN^- standard. The units of concentration for the CN^- standards is in µg.

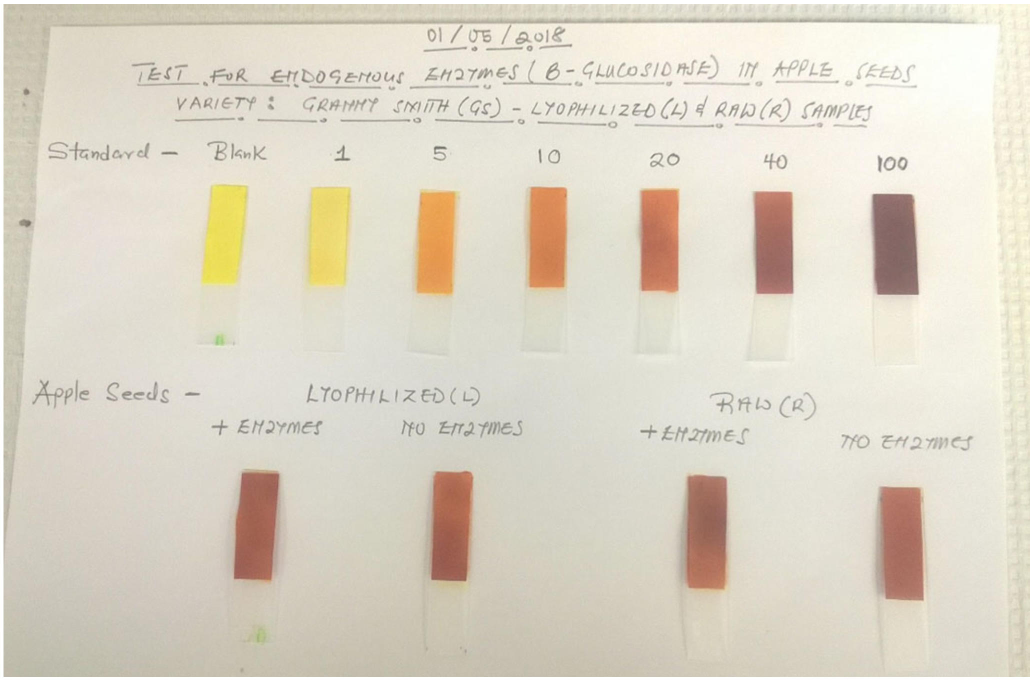

Figure S7. Picrate-paper results for endogenous enzymes test for raw (fresh) and lyophilized apple seeds. The top row of strips indicates the color changes for different concentrations of amygdalin (µg) as CN^- standard. The units of concentration for the CN^- standards is in µg.

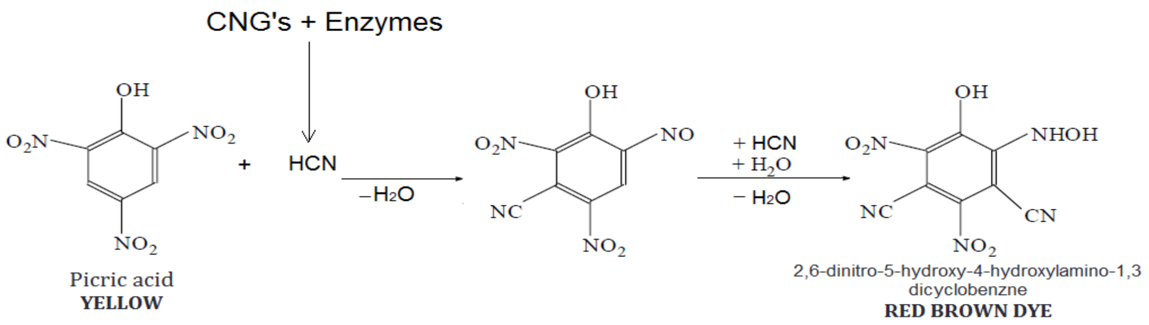

Figure S8. Cyanide reaction with picric acid.
